# A Mechanism-Based Model for the Prediction of the Metabolic Sites of Steroids Mediated by Cytochrome P450 3A4

**DOI:** 10.3390/ijms160714677

**Published:** 2015-06-30

**Authors:** Zi-Ru Dai, Chun-Zhi Ai, Guang-Bo Ge, Yu-Qi He, Jing-Jing Wu, Jia-Yue Wang, Hui-Zi Man, Yan Jia, Ling Yang

**Affiliations:** 1 Dalian Institute of Chemical Physics, Chinese Academy of Sciences, Dalian 116023, China; E-Mails: daiziru@dicp.ac.cn (Z.-R.D.); aicy@dicp.ac.cn (C.-Z.A.); geguangbo@dicp.ac.cn (G.-B.G.); hyqjeff@outlook.com (Y.-Q.H.); wjj@dicp.ac.cn (J.-J.W.); wangjy@dicp.ac.cn (J.-Y.W.); dldxmanhuizi@163.com (H.-Z.M.); jiayan@dicp.ac.cn (Y.J.); 2 Graduate School of Chinese Academy of Sciences, Beijing 100049, China

**Keywords:** CYP3A4, steroids, metabolic site, mechanism-based prediction, activation energy

## Abstract

Early prediction of xenobiotic metabolism is essential for drug discovery and development. As the most important human drug-metabolizing enzyme, cytochrome P450 3A4 has a large active cavity and metabolizes a broad spectrum of substrates. The poor substrate specificity of CYP3A4 makes it a huge challenge to predict the metabolic site(s) on its substrates. This study aimed to develop a mechanism-based prediction model based on two key parameters, including the binding conformation and the reaction activity of ligands, which could reveal the process of real metabolic reaction(s) and the site(s) of modification. The newly established model was applied to predict the metabolic site(s) of steroids; a class of CYP3A4-preferred substrates. 38 steroids and 12 non-steroids were randomly divided into training and test sets. Two major metabolic reactions, including aliphatic hydroxylation and *N*-dealkylation, were involved in this study. At least one of the top three predicted metabolic sites was validated by the experimental data. The overall accuracy for the training and test were 82.14% and 86.36%, respectively. In summary, a mechanism-based prediction model was established for the first time, which could be used to predict the metabolic site(s) of CYP3A4 on steroids with high predictive accuracy.

## 1. Introduction

Identification of the metabolic pathways of a given compound is important for the lead optimization in the early stage of drug discovery and development [1]. As the most abundant P450 isoform expressed in human liver, cytochrome P450 3A4 (CYP3A4) catalyzes nearly 50% of the therapeutic drugs currently in clinical use [2]. It is well known that CYP3A4 displays a weak substrate specificity and poor regioselectivity, due to its large active cavity, which makes it a huge challenge to predict the metabolic site(s) on CYP3A4 substrates, especially for those compounds with rigid structures [3,4]. Among all of the reported CYP3A4 substrates, endogenous steroidal hormones and steroid drugs have attracted much attention, since they play vital roles in various physiological and pharmacological processes in the endocrine system, neuronal system and the immune system [5,6]. However, previous studies demonstrated that the metabolic types and the metabolic site(s) of steroids are numerous and complex. For example, CYP3A4 prefers to catalyze 6-hydroxylation on steroids with 3-keto-4-ene, such as testosterone and medroxyprogesterone acetate (MPA), due to the pharmacophoric features and the electronic reactivity of H atoms at the C-6 site [7,8,9,10,11]. Cholesterol and dehydroepiandrosterone (DHEA), two 3-β-hydroxy-5-ene steroids, are preferentially hydroxylated at the C-4 and the C-7 position, respectively [12,13]. Moreover, in addition to aliphatic hydroxylation, CYP3A4 also catalyzes N-dealkylation of antiprogestins [14,15]. The varied metabolic reactions and the multiple potential metabolic sites within steroids, combined with the complex conformation of CYP3A4, make it hard to predict the metabolic sites of CYP3A4 with an *in silico* approach.

To date, two classes of *in silico* models, ligand-based and structure-based approaches, have been proposed to predict the regioselectivity of CYP substrates [16]. In ligand-based approach, structures of known active and inactive compounds were used to establish the structure-activity relationships, while the metabolic reactions were extracted from the existing databases. Now, four ligand-based models, including rule-based methods [17], pharmacophore-based methods [18], quantitative structure activity relationship (QSAR) methods [19] and reactivity-based *ab initio* calculations [20,21,22] are widely used to explore the potential metabolic sites. Among all input parameters in these above-mentioned methods, chemical reactivity is the key determinant, which is assumed as the rate-determining step of a metabolic reaction. Singh *et al*. estimated hydrogen abstraction energies by AM1 molecular orbital calculations and predicted the metabolic sites by the energies and surface area of the hydrogen atoms with high accuracy [20]. Although the prediction methods based on a ligand-based approach consume less time, the information of substrate reactivity and the shape of active sites were not considered. Thus, they fail to predict the regioselectivity with rational results [23]. In contrast, structure-based approaches focus on the properties of a given enzyme and its interactions with the ligand, as well as the reaction mechanism [24]. These structure-based models utilize docking-based approaches to predict the metabolic site(s) of known substrates based on the binding mechanism of compounds in the active site of each CYP enzyme [25]. However, CYP enzymes exhibit extraordinary levels of flexibility, and the structure-based algorithms are highly susceptible, even to marginal conformational changes of the target enzyme, which might become a major problem for structure-based approaches in general [26].

Compared with other CYP3A substrates, steroids have a rigid skeleton and many possible metabolic sites catalyzed by the CYP enzyme; thus, it is very difficult to predict the metabolic site(s) of steroids by simply using currently available algorithm and models. There are at least two challenges in prediction of the metabolic site(s) on CYP3A4-mediated steroids’ metabolism with high accuracy. First, most steroids have nearly identical rigid skeleton, and their structures are too similar to change or adjust their conformations into the active site of CYP3A4. Second, the substrate binding cavity of CYP3A4 is so large that the substrate can be easily docked into the active cavity with varied conformation types and thus generates different metabolic sites. In these cases, the development of a novel prediction model is highly desirable. It is well known that the metabolic process includes the binding and the catalytic processes. The binding process decides the best-fitting conformation, while the catalytic process determines the difficulty of the catalysis. Generally, the selectivity is tightly associated with the substrate binding pocket, in which enzymes catalyze the eventual reactions. Different types of reactions catalyzed by heme (especially for hydroxylation) follow a two-step mechanism, in which the hydrogen abstraction step possesses the highest energy barrier [27,28,29]. Recent investigations have also indicated that the activation energy is directly correlated to hydrogen abstraction reaction energy [28]. Therefore, the activation energy barrier of the transition state in a catalytic process could represent the difficulty of the reaction.

The aim of this study was to predict the metabolic site(s) of CYP3A4-mediated biotransformation of steroids and other typical substrates. To this end, a novel mechanism-based prediction model was developed on the basis of the binding conformation and the molecular reactivity, which was used to reveal the process of real metabolic reactions and the site(s) of modification. The methods for the generation of the bioactive conformations and the hydrogen abstraction energy estimation were implemented to predict the accessibility of each atom of the substrate toward the heme pocket of CYP3A4, as well as their corresponding reactivity. Taking into account both accessibility and reactivity in the prediction model, the binding and the catalytic process could be well simulated, thus making better predictions of the metabolic site(s) of various CYP3A4 substrates with high accuracy.

## 2. Results and Discussion

### 2.1. Analysis of Active Conformations

In order to predict the metabolic site(s) of steroids in CYP3A4, the binding accessibility and the activation energy of metabolic reaction(s) were combined together to establish a mechanism-based model. The active conformations were obtained by using the package (Surflex-Dock) to generate 50 possible binding orientations of each substrate. Before docking studies, several representative CYP3A4 crystal structures were selected and compared. CYP3A4 with ritonavir (coded as 3TJS) [30] was ultimately selected for the generation of active conformations, due to its best docking results for 10 typical substrates in the training set. Then, all of the 50 CYP3A4 substrates were docked into this CYP3A4 crystallographic structure, while the CYP3A4 crystal structure was treated as rigid yet substrates as flexible.

It is well known that the ligand can bind with the target enzyme in various conformations. However, for a given CYP3A substrate, the distance between the potential metabolic sites and the heme of CYP3A4, as well as the binding affinity of this compound towards target enzyme are the most important factors affecting CYP3A4-mediated site-specific biotransformations. Thus, the distances (*r*) between the heme iron of CYP3A4 and the potential metabolic site(s) of each substrate, as well as the binding affinity-related parameters were used to predict the preferred metabolic site(s) of CYP3A4 substrates in this study. Firstly, the distances (*r*) between the heme iron of CYP3A4 and the potential metabolic site(s) of each tested substrate were obtained. We selected the top ten bioactive poses from the 50 solutions according to the value of the ChemScore fitness function and calculated the distance between the preferred site and the heme iron [24]. To increase the possibility of finding out the correct metabolic site(s) of the substrate in CYP3A4, a bond length adjacent to the preferred site was also accounted for as a potential metabolic site. Finally, the predictability of a potential metabolic reaction site was calculated as the weighted fraction of docking poses with a site-heme distance ranging from 2.5 to 7.5 Å. Secondly, hydrogen bonding and lipophilic interactions for the top ten bioactive poses of each substrate were calculated and served as the key interaction parameters to evaluate the binding affinity, for hydrogen bonding and lipophilic interactions are two key determinants for P450 substrate selectivity and binding affinity [31].

**Figure 1:**
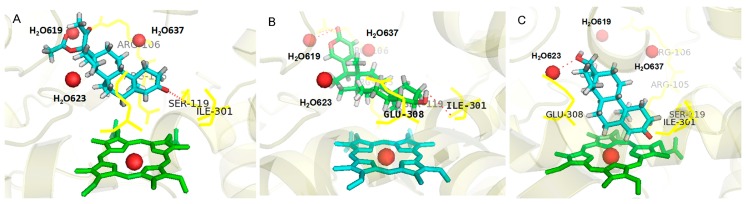
The binding modes of three typical substrates docked into the active cavity of CYP3A4 via hydrogen bond interactions; (**A**) medroxyprogesterone acetate (MPA); (**B**) bufalin (BF); and (**C**) testosterone. The important residues interacting with the bound substrate in the CYP3A4 complex are shown as yellow lines; the three water molecules are shown as red spheres; and the heme groups are shown as ball-and-stick.

As shown in Figure 1A and the docking results (Tables S1 and S2), more than half of all of the tested substrates can form the hydrogen bonds with Arg105, Arg106, Ser119, Ile301 and Glu308, some forming the bonds with benzene ring carbonyl oxygen, like medroxyprogesterone acetate (MPA), while others bind at the benzene ring hydroxyl of the ligand, like bufalin (BF) (Figure 1B). In addition, more than a quarter of substrates formed a hydrogen bond with water molecules H_2_O-619, H_2_O-623 and H_2_O-637, like testosterone (Figure 1C). It can be speculated that H-bond served as an important force in driving CYP3A substrates to adopt the right conformation in the active cavity, which can also explain why almost all metabolic sites located on the atom adjacent to benzene ring carbonyl oxygen or hydroxyl sites. Thus, the key hydrogen-bond donors, including five amino acids in CYP3A4 and the water molecule, were considered as crucial features to establish the prediction models.

**Figure 2:**
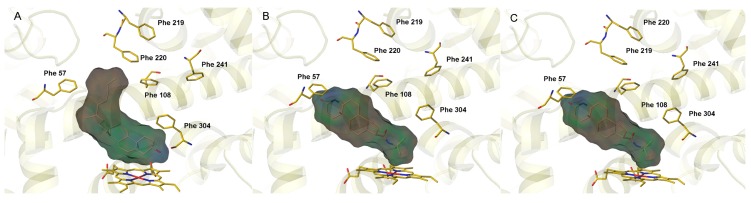
The binding modes of three typical substrates docked into the active cavity of CYP3A4 via lipophilic interactions with Phe-clusters or the heme. (**A**) Cholesterol; (**B**) finasteride; and (**C**) pregnenolone. The important residues interacting with the bound substrate in the CYP3A4 complex and a heme group are shown as yellow capped sticks.

Compounds, like cholesterol and finasteride, tended to perform hydrophobic interactions with phenylalanine clusters at the roof of the active pocket. Phe-57, Phe-108, Phe-219, Phe-220, Phe-241 and Phe-304 were found exposed to the surrounding active site and formed the hydrophobic cluster interacting with some substrates. As shown in Figure 2A,B, several substrates containing lipophilic substitutes located in the active pocket of CYP3A4 via lipophilic interaction with Phe-clusters. For those substrates without lipophilic interaction with hydrophobic clusters, their metabolic site(s) happened to be hydrophobic toward the heme, just like pregnenolone (Figure 2C). The hydrophobic interactions was thus considered as an increased force of a ligand binding with the surface of the active cavity and selected as another key feature to build the mechanism-based prediction model. On the basis of the site-heme distance and key interaction features, 367 potential metabolic sites were selected and then used to evaluate the molecular activation energy.

### 2.2. Estimation of Activation Energy

For each potential metabolic site, the reactivity was further evaluated by calculating the activation energy following a two-step catalysis mechanism, which was directly correlated to hydrogen abstraction energy. Here, the reactivity for potential hydrogen atoms was calculated with the Complete Neglect of Differential Overlap (CNDO) semi-empirical method. A total number of 50 substrates involving two major metabolic reactions (aliphatic hydroxylation and *N*-dealkylation) have been evaluated, with 1005 nonequivalent hydrogen atoms attached to carbon. Regardless of the binding process, the reactivity evaluation exhibited a success rate of 62.00% (Table 1) and varied in accordance with the type of metabolic reaction. Notably, *N*-dealkylation achieved the highest prediction accuracy up to 80.00%, while aliphatic hydroxylation had relatively lower predictability of 57.50%.

Given that CYP3A4 had a relatively large active site cavity and broad substrate specificity, the model can hardly predict CYP3A4-mediated metabolism with high accuracy with the reactivity alone.

**Table 1:** Prediction accuracy for CYP3A4 substrates using the top three sites for the activation energy estimation.

Reaction Types	Model of Activation Energy Estimation		
	Nc ^a^	N ^b^	Ratio (%) ^c^
Aliphatic hydroxylation	23	40	57.50%
*N*-dealkylation	8	10	80.00%
Total	31	50	62.00%

Nc ^a^ = Number of correctly predicted substrates; N ^b^ = Number of substrates; ^c^ Percentage of correct predictions.

### 2.3. Application of the Mechanism-Based Model

Since the reactivity evaluation alone can hardly achieve rational accuracy for metabolic site prediction, both the binding conformation and the reaction activity of substrates are used to establish a mechanism-based prediction model. Active conformations were generated based on the representative features and the cutoff distance to recognize the potential sites. Subsequently, those potential metabolic sites were ranked based on their activation energy, with the top one treated as the most possible metabolism site. The training set included 28 steroids, and 255 potential sites were recognized. As shown in Table 2, in the training set, 82.14% known metabolic sites that have been confirmed by experiment are listed in the top three predicted sites by using this mechanism-based model. Particularly, all *N*-dealkylation metabolic sites have been accurately predicted; also, the predictability of aliphatic hydroxylation metabolic sites reached 80%.

**Table 2:** Prediction accuracy for the training set using the top three sites for themechanism-based prediction model.

Reaction Types	Training Set		
	Nc ^a^	N ^b^	Ratio (%) ^c^
Aliphatic hydroxylation	20	25	80.00%
*N*-dealkylation	3	3	100%
Total	23	28	82.14%

Nc ^a^ = Number of correctly predicted substrates; N ^b^ = Number of substrates; ^c^ Percentage of correct predictions.

In order to further validate the universality of this newly developed prediction model, we expanded the structure types of CYP3A4 substrates. Ten steroids and 12 non-steroids CYP3A4 substrates with varied skeletons and 112 potential metabolic sites were selected in the test set, while *N*-dealkylation and aliphatic hydroxylation were also involved. As shown in Table 3, the predictability of the metabolic sites undergoing aliphatic hydroxylation was also up to 80% in the test set, while the predictability of the metabolic sites undergoing *N*-dealkylation was very high, up to 100%. The overall forecast of the test set was 86.36%. Overall, the newly established model exhibited good prediction accuracy for *N*-dealkylation and aliphatic hydroxylation of CYP3A4 substrates. These findings also suggested that it is necessary to evaluate the binding accessibility and activation energy simultaneously together, for the precise prediction of the metabolic site(s) of substrates in CYP3A4.

**Table 3:** Prediction accuracy for the test set using the top three sites for the mechanism-based prediction model.

Reaction Types	Test Set		
	Nc ^b^	N ^a^	Ratio (%) ^c^
Aliphatic hydroxylation	12	15	80.00%
*N*-dealkylation	7	7	100%
Total	19	22	86.36%

N ^a^ = Number of substrates; Nc ^b^ = Number of correctly predicted substrates; ^c^ percentage of correct predictions.

### 2.4. Analysis of the Prediction Model

Metabolism will cause qualitative transformation in the structures of drug molecules, which, in turn, causes the changes in pharmacokinetic and toxicological effects. As the most important human drug-metabolizing enzyme, CYP3A4 has a large and flexible active cavity and catalyzes structurally diverse substrates, such as steroids, macrolides, dibenzocyclooctadiene lignans and benzodiazepines. The weak substrate specificity and poor regioselectivity make it difficult to predict the metabolic site(s) on its substrates, especially for steroids with rigid structures, using the existing algorithms. In this study, a new mechanism-based model was established for the precise prediction of the metabolic site(s) of substrates in CYP3A4. To this end, 38 steroids and 12 non-steroids compounds with known metabolic sites in CYP3A4 were used to investigate and validate the accuracy and the universality of this newly developed prediction model.

During the processes of model construction, binding affinity and activation energy were combined together to predict the accessibility of each substrate towards the target enzyme, as well as their corresponding reactivity. Our results demonstrated that the newly developed prediction model could not only predict the metabolic sites of steroids with high accuracy (84.21%), but also be applied to other CYP3A4 substrates with considerable accuracy (91.66%). Notably, the predictability of the mechanism-based model was obviously higher compared with former models based on a single approach, either structure-based or ligand-based. For example, the structure-based approach proposed by Poongavanam *et al*. has a predictability of only 55% on active compounds and 75% on the inactive compounds [32], while the ligand-based approach proposed by Singh *et al*. has a predictability of 78% [32].

CYP3A4 catalyzes a broad range of metabolic reactions, such as hydroxylation, oxidation and *N*-dealkylation. For natural steroids and their synthesized analogous, aliphatic hydroxylation and *N*-dealkylation are the two most important metabolic reaction types for these compounds in CYP3A4; thus, it is highly desirable to precisely predict the metabolic site(s) of steroids undergoing these two metabolic reactions [33,34]. In this study, our established model could predict the metabolic sites of steroids with high accuracy, which should be attributed to the reactivity of the above two reactions which is highly relevant to hydrogen atom energy. The reaction mechanism of aliphatic hydroxylation is a “hydrogen abstraction/oxygen rebound mechanism” [35]. While for CYP-mediated *N*-dealkylation, there are two alternative mechanisms: One is hydrogen atom transfer, and the other is single electron transfer. All mechanisms reflect reactivity and the hydrogen atom energy of the aliphatic hydroxylation and *N*-dealkylation metabolic reaction are highly correlated; The barriers of the *N*-dealkylation hydrogen-atom transfer reaction are much lower than those of aliphatic hydroxylation, which can explain why the predictability of *N*-dealkylation is higher than that of aliphatic hydroxylation [36,37].

## 3. Experimental Section

### 3.1. Dataset and Molecule Modeling

**Figure 3:**
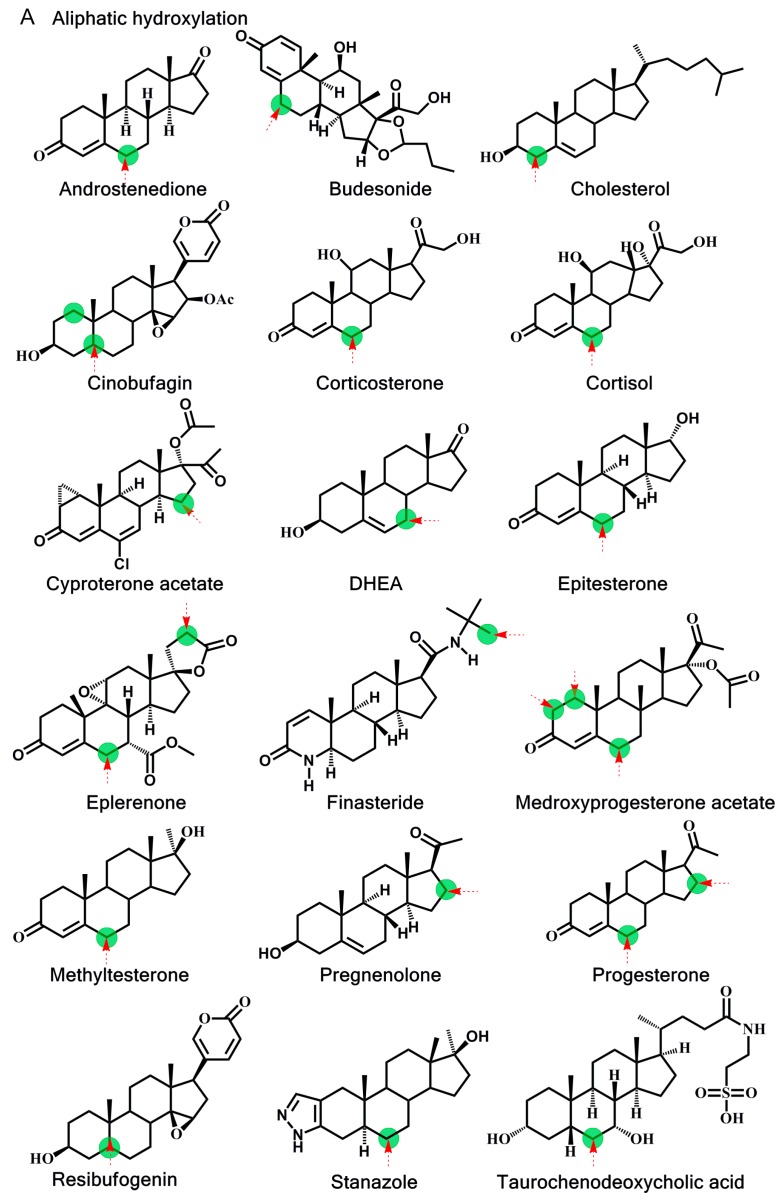

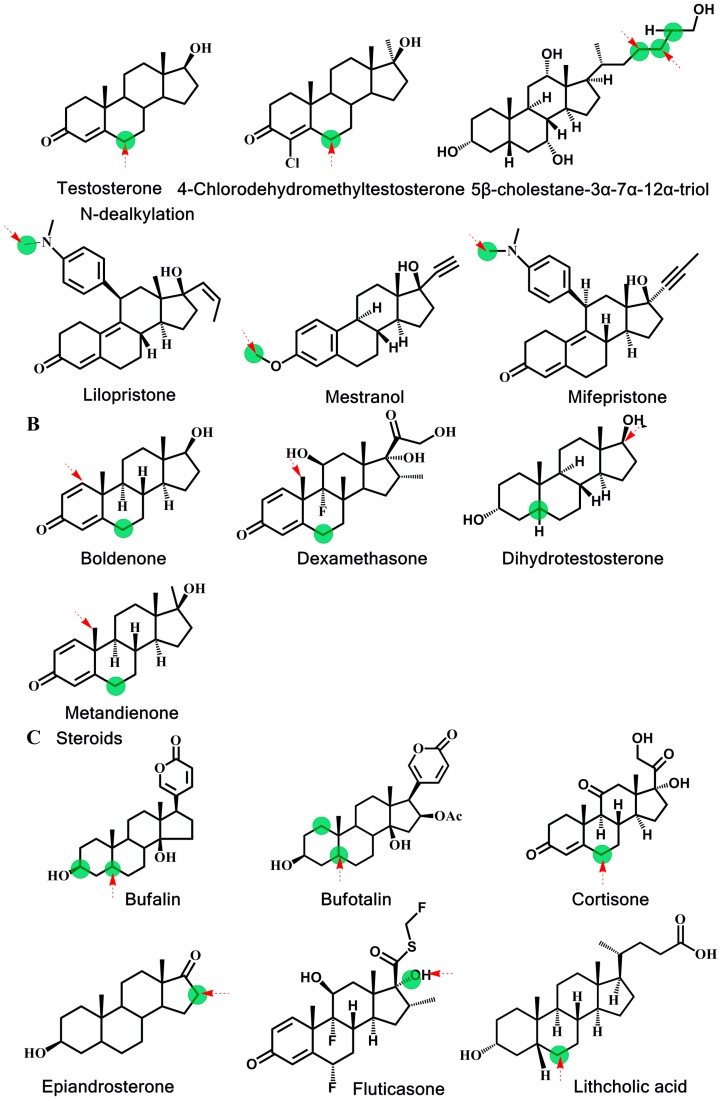

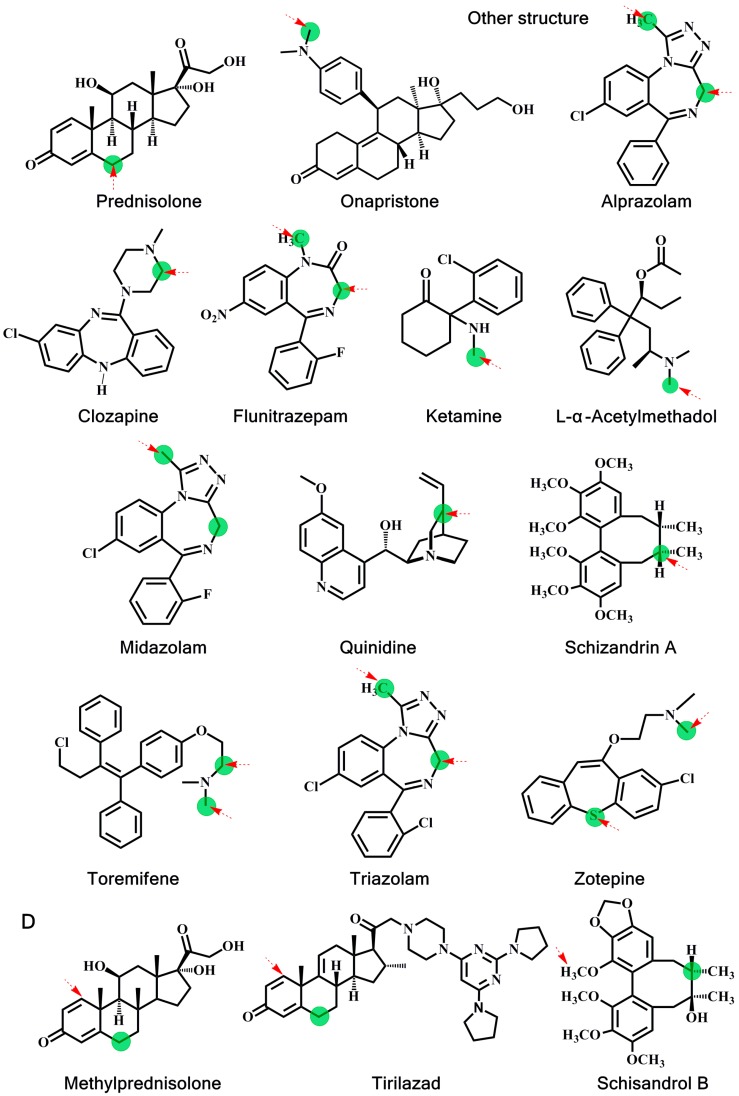
Chemical structures of: (**A**) Successful cases in the training set; (**B**) Failed cases in the training set; (**C**) Successful cases in the test set; and (**D**) Failed cases in the test set. Experimentally known major sites of metabolism are indicated with a green circle. Predicted sites with the mechanism-based prediction model are indicated with an arrow. DHEA, dehydroepiandrosterone.

Metabolic reaction data were collected from the literature [9,20,38,39,40,41]. Two major metabolic reactions of steroids mediated by CYP3A4, aliphatic hydroxylation and *N*-dealkylation were included in the dataset. The set of 50 substrates was divided into two set, with 28 substrates belonging to the training set and 22 substrates (included steroids and other structures) classified in the test set. In the total of 50 collected substrates, 37 of them were reported to have only one metabolite, whereas another 13 to have more than one metabolite in the CYP3A4-mediated metabolism [14,20,38]. The training set was applied for model construction, and the test set was applied to the prediction model validation. The set of 50 CYP3A4 substrates used in our study is shown in Figure 3.

Molecular energy minimization was carried out by the standard Tripos force with delta energy change 0.05 kcal/mol energy convergence criteria, and the most stable conformation was searched through the Powell conjugate gradient algorithm with a convergence criterion of 0.001 kcal/mol. The energy gradient threshold value was set at 0.05 kcal/mol/Å.

### 3.2. Generating Active Conformations

Surflex-Dock, the incremental construction-based docking method, has been applied to generating active conformations by exploring all calculations of binding interactions and conformations of ligands interfaced with Sybyl 7.3 (Tripos, St. Louis, MO, USA) [42]. Surflex-Dock uses an empirical scoring function and a patented search engine to generate the bioactive binding poses of substrates in the active site of CYP3A4. During the docking simulation, the enzyme structure was kept rigid, while the substrate was left fully flexible by changing the conformations of the ligand in the active site. Here, several representative X-ray crystallographic structures of human microsomal CYP3A4 bound in the Protein Data Bank were selected for the docking study, including CYP3A4, coded as 1TQN, CYP3A4 with progesterone, coded as 1WOE, and CYP3A4 with ritonavir, coded as 3TJS [43,44]. CYP3A4 with ritonavir was eventually selected for the active conformations evaluations, because it gave the best docking results when tested on ten typical substrates in the training set. The active site was defined as a 2.25-Å radius circles around residues Phe57, Arg105, Arg106, Phe108, Ser119, Ile120, Ile301, Glu308, Leu482 and Leu483. In view of water molecules participating in the interaction between the substrate and CYP450s, crystallographic water molecules were considered in this study [45]. The charge assignment was set as formal, and the orientations were evaluated using the ChemScore function. The best bioactive poses selected from the top ten solutions were then used for the analysis of the binding interactions between CYP3A4 and its substrates.

### 3.3. Activation Energy Estimation

The models proposed by Singh were used to represent the reactivity of labile sites within a substrate. In the model, the reactivity of a hydrogen atom within a substrate was treated as the reaction energy of hydrogen abstraction. The energy to extract the hydrogen atom on an isolated substrate is given by:

Δ*H_rxn_=* Δ*H*_2_*−* Δ*H*_1_

The hydrogen abstraction reaction energy (Δ*H_rxn_*) is given by the difference between the heat of the formation of the native substrate (Δ*H*_1_) and that of its radical (Δ*H*_2_). Calculations of hydrogen were carried out on the basis of a semi-empirical force field with the HYPERCHEM 7.5 program (Hypercube, Gainsville, FL, USA). Each molecule was constructed using the SYBYL sketcher, assigned Gasteiger–Hückel charges and relaxed using the Tripos force field. Then, they were optimized by the CNDO semi-empirical method. Single-point energy was also carried out using the CNDO semi-empirical method. Default parameters available in the HYPERCHEM 7.5 package were used for all of the calculations. In hydrogen abstraction reactions, hydrogen atoms attached to the same carbon atom have the same radical species in a metabolic reaction.

### 3.4. Prediction Models

**Figure 4:**
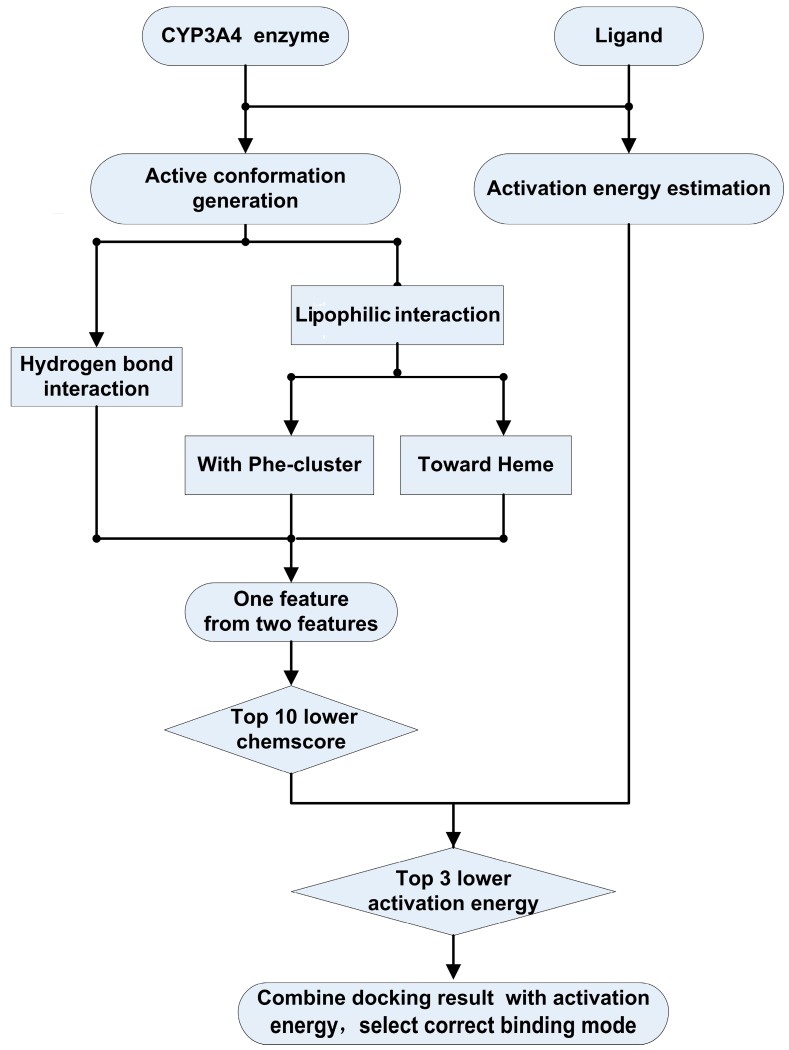
The workflow of the mechanism-based prediction model.

In consideration of both the binding and catalysis process, binding accessibility and activation energy were prepared to develop the model for the prediction of metabolic sites (Figure 4).

**Figure 5:**
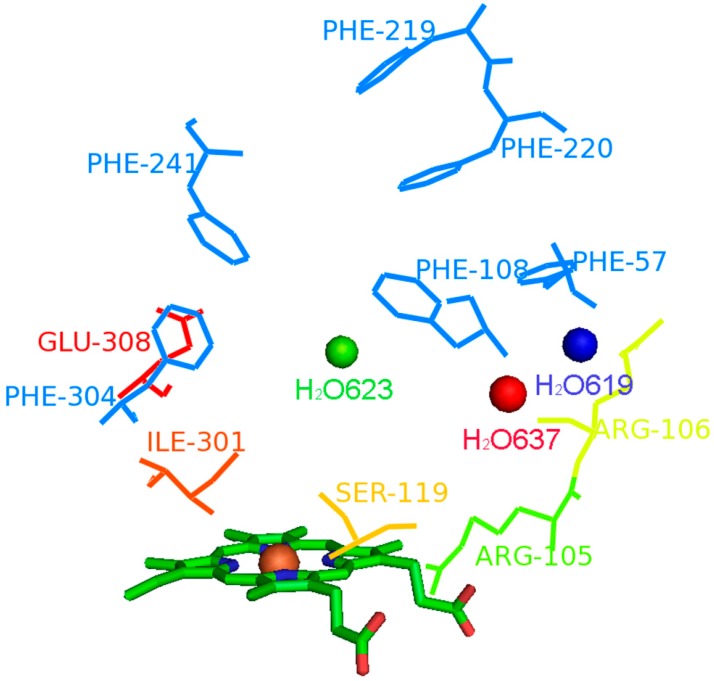
Representative interaction features in CYP3A4 active conformation. Shown are six hydrogen bonds (three water as spheres and three residues as magenta lines), six hydrophobic features (blue line) and a heme (ball-and-stick).

The potential sites of the substrates can be determined from the following aspects:

1. The distances (*r*) between the heme iron of CYP3A4 and the atoms of the substrates [21]: A catalytically-reactive distance from the heme iron of CYP450 is generally known to be within 6 Å; however, the range of distances used in this model was rather large due to ligand-induced conformational changes of the active site [26], for the CYP3A4 crystal structure exhibits a relatively large substrate-binding cavity volume [31]. Thus, the results from dockings with three different distance cutoffs (6.5, 7.0 and 7.5 Å distance from the heme iron atom to the carbon atom in the substrate) on the 28 substrates in the training set were evaluated [46]. Additionally, a docking score with a cutoff value of 7.5 Å for our combined evaluations has been selected, because it gives the best docking results when considering the top ten ranked sites and it also sets the pose within the cutoff for at least one major metabolite. It is worth considering that the sites adjacent to the speculated metabolic sites were also considered as potential metabolic sites.

2. Key interaction features: It is well known that the position of the metabolic site was determined based on the CYP3A4 active conformations and was confirmed by visual inspection. CYP3A4 substrates are usually of high volume, relatively lipophilic and structurally diverse with one or two hydrogen bond donors/acceptors nearby the site of metabolism [47]. Therefore, the features of a metabolic site were described by hydrogen bond interactions and lipophilic interactions [48], which were assumed to be fundamentally important to P450 substrate selectivity and binding affinity, moreover having an impact on not only the conformation selection, but also the substrate orientation [49]. Based on the training set of steroid substrates, two representative interaction features having the maximum number of substrates with a correct binding mode were selected (Figure 5): (1) Hydrogen-bond donor (5 key amino acids and 3 vital water molecule); and (2) Lipophilic interaction (lipophilic interaction with hydrophobic Phe-cluster or toward the heme).

For a comprehensive forecast, the conformations satisfying site-heme distance and one of the key features were considered as potential sites, which were further ranked by the activation energy to predict metabolic sites. Since the calculation method in the model contains inaccuracy, if the experimental major metabolic site were coincident with one of the top three rank positions, it was supposed that the metabolic sites’ prediction was correct. For those substrates with multiple metabolic sites, as long as one predicted metabolic site is listed in the top three positions, this will be considered as a correct prediction. To ascertain the accuracy of our prediction model, the forecast results were compared with the experimental results. The model was validated by a test set with steroid compounds, as well as other substrate structures.

## 4. Conclusions

In this study, a comprehensive mechanism-based prediction model combining binding accessibility and activation energy estimation was established to predict the metabolic sites of steroids in CYP3A4 for the first time. A dataset of 50 CYP3A4 substrates and two major metabolic reactions, including aliphatic hydroxylation and *N*-dealkylation, were involved. The established model reliably identified at least one observed metabolic site of these substrates in the top three positions with an accuracy of 82.14% for the training set and 86.36% for the test set. All of these data suggested that the newly established mechanism-based prediction model displayed high predictive accuracy, which could be used to predict rigid substrates, such as steroids, as well as other CYP3A substrates with varied skeletons.

## Supplementary Materials

Supplementary materials can be found at http://www.mdpi.com/1422-0067/16/07/14677/s1.
