# Supplementary material for: A Mechanism-Based Model for the Prediction of the Metabolic Sites of Steroids Mediated by Cytochrome P450 3A4
Source: Int J Mol Sci. 2015 Jun 30;16(7):14677–94. doi: 10.3390/ijms160714677 (PMC4519866; doi:10.3390/ijms160714677)
Supplement: Supplementary file 1 [file ijms-16-14677-s001.pdf]

Supplementary Information

Table S1. Original parameter of docking data for the compounds from training set.

Rank	Distance	Key Interaction Features			Chemscore	Predicted Metabolic Sites	Actual Metabolic Sites
		Hydrogen Bond Interaction	Lilophilic Interaction toward Heme	Lilophilic Interaction with Phe−Cluster			
**Androstenedione**							
1	2.04	−	−	−	−31.4616	3	6-Hydroxylation
2	6.67	H_2O619 Ser119	+	+	−30.8448	6	
3	2.45	−	+	+	−29.0663	16	
4	9.03	−	−	−	−28.568	7	
5	2.71	−	+	+	−28.321	16	
6	8.83	−	−	−	−27.9362	7	
7	8.96	−	−	−	−27.3553	10-Methyl	
8	9.52	−	−	−	−27.0542	10-Methyl	
9	4.07	−	+	−	−26.5437	16	
10	9.32	−	−	−	−25.3265	7	
**Boldenone**							
1	3.66	H_20637	−	−	−31.5316	3	6-Hydroxylation
2	9.65	−	−	−	−31.2291	10-Methyl	
3	2.75	−	+	−	−28.3998	2	
4	5.76	−	−	+	−28.3105	15	
5	4.34	Arg105 Ala305 Ile301	+	−	−25.634	12	
6	3.89	H_20637	−	−	−25.5377	3	
7	10.32	−	−	−	−24.353	7	
8	6.76	−	−	−	−23.5347	16	
9	3.04	−	+	+	−22.6789	6	
10	10.56	−	−	−	−21.8564	7	

Table S1. *Cont.*

Rank	Distance	Key Interaction Features			Chemscore	Predicted Metabolic Sites	Actual Metabolic Sites
		Hydrogen Bond Interaction	Lilophilic Interaction toward Heme	Lilophilic Interaction with Phe−Cluster			
				**Budesonide**			
1	2.18	H_2O623	−	+	−37.2385	27	6-Hydroxylation
2	2.96	−	−	−	−36.8346	25	
3	3.89	Arg105 GLu374	+	−	−36.538	3	
4	3.08	−	−	−	−36.4359	25	
5	3.54	Arg105	+	−	−36.394	3	
6	3.59	Arg105 GLu374	+	−	−36.3256	3	
7	3.95	−	−	−	−36.2559	3	
8	2.32	H_2O623	−	+	−36.163	27	
9	4.28	H_20637	+	+	−36.1132	6	
10	4.23	Arg105 GLu374	+	−	−36.0791	3	
				**Cholesterol**			
1	2.3	H_2O637 H_2O619 Arg372	−	+	−39.8893	23	4-Hydroxylation
2	2.35	H_2O619 Arg372 heme	−	−	−39.4872	26	
3	3.77	−	−	−	−39.3215	23	
4	2.38	−	−	−	−39.3057	26	
5	3.12	H_2O619 Arg372	−	+	−39.1173	24	
6	9.38	−	−	−	−39.0149	7	
7	3.91	−	−	−	−38.8462	23	
8	3.04	−	+	+	−38.7725	4	
9	4.6	−	−	−	−38.5956	23	
10	10.08	−	−	−	−38.5865	7	

Table S1. *Cont.*

Rank	Distance	Key Interaction Features			Chemscore	Predicted Metabolic Sites	Actual Metabolic Sites
		Hydrogen Bond Interaction	Lilophilic Interaction toward Heme	Lilophilic Interaction with Phe−Cluster			
				**Cinobufagin**			
1	6.32	Glu308	−	+	−33.4728	5	1,5-Hydroxylation
2	5.7	−	−	+	−33.2657	5	
3	3.96	Heme	−	−	−33.0704	3	
4	3.07	−	+	−	−32.7474	16-Benzene ring	
5	8.25	−	−	−	−32.7373	10-Methyl	
6	4.21	Heme	−	−	−32.5876	3	
7	3.53	−	+	−	−32.4844	16-Benzene ring	
8	8.76	−	−	−	−32.4595	13-Methyl	
9	4.65	−	−	−	−32.2311	3	
10	9.25	−	−	−	−32.1357	10-Methyl	
				**Corticosterone**			
1	1.99	Leu210	−	−	−38.8151	3	6-Hydroxylation
2	3.9	Leu210	−	−	−37.6235	3	
3	3.91	−	−	−	−36.2652	3	
4	2.18	H_20637 heme	−	−	−34.7941	20	
5	3.94	Leu210	−	−	−34.6832	3	
6	3.21	H_20637 heme	−	−	−34.2786	20	
7	5.62	Arg105 Glu308 Gln484	−	−	−34.1306	6	
8	5.18	H_20637	−	−	−33.7562	20	
9	7.25	Heme	−	−	−33.4578	20	
10	7.64	H_20637 heme	−	−	−33.1325	20	

Table S1. *Cont.*

Rank	Distance	Key Interaction Features			Chemscore	Predicted Metabolic Sites	Actual Metabolic Sites
		Hydrogen Bond Interaction	Lilophilic Interaction toward Heme	Lilophilic Interaction with Phe−Cluster			
**Cortisol**							
1	6.46	−	+	−	−33.9684	17	6-Hydroxylation
2	6.49	−	+	−	−33.2572	17	
3	6.85	−	−	−	−32.5628	17	
4	8.23	−	−	−	−31.7041	7	
5	6.79	−	+	−	−31.3267	17	
6	4.09	−	−	+	−31.0709	6	
7	4.23	−	−	+	−30.8649	6	
8	7.23	−	−	−	−30.695	18	
9	7.24	−	−	−	−30.3425	18	
10	8.03	−	−	−	−30.125	18	
**Cyproterone Acetate**							
1	6.87	−	+	+	−35.968	15	15-Hydroxylation
2	9.51	−	−	−	−35.6617	10-Methyl	
3	5.11	H_2O637	−	−	−35.4992	3	
4	928	−	−	−	−35.1236	7	
5	5.24	−	+	+	−34.9375	3	
6	10.25	−	−	−	−34.6228	10-Methyl	
7	9.87	−	−	−	−33.8329	10-Methyl	
8	10.63	−	−	−	−33.1345	10-Methyl	
9	10.08	−	−	−	−32.6743	7	
10	10.35	−	−	−	−32.4228	7	

Table S1. *Cont.*

Rank	Distance	Key Interaction Features			Chemscore	Predicted Metabolic Sites	Actual Metabolic Sites
		Hydrogen Bond Interaction	Lilophilic Interaction toward Heme	Lilophilic Interaction with Phe−Cluster			
**Dexamethasone**							
1	5.28	Arg105	−	+	−30.6036	6	6-Hydroxylation
2	5.63	Arg105	−	+	−30.5376	6	
3	4.43	H_2O637 Glu74	−	+	−30.4537	4	
4	5.54	Arg105	−	−	−30.2758	6	
5	4.54	H_2O637 Glu74	−	+	−30.1045	4	
6	3.68	H_2O637	−	−	−29.9487	2	
7	5.94	−	−	+	−29.753	6	
8	6.09	Arg105	−	+	−29.6432	6	
9	5.83	−	−	−	−29.5415	4	
10	2.48	−	−	−	−29.4292	22	
**DHEA**							
1	7.79	Ser119 Val240	−	−	−38.1107	10-Methyl	7-Hydroxylation
2	5.53	Ser119	−	−	−36.3542	10-Methyl	
3	7.92	Ser119 Val240	−	−	−34.9812	10-Methyl	
4	8.05	Ser119 Glu308	+	+	−33.1552	7	
5	7.98	−	−	−	−31.8528	3	
6	8	Ser119 Val 240	−	−	−30.4589	10-Methyl	
7	8.12	−	−	−	−29.6338	3	
8	4.81	Arg372	−	+	−28.8657	13	
9	3.04	Arg106 Glu374	+	+	−28.7962	16	
10	8.23	−	−	−	−27.8442	3	

Table S1. *Cont.*

Rank	Distance	Key Interaction Features			Chemscore	Predicted Metabolic Sites	Actual Metabolic Sites
		Hydrogen Bond Interaction	Lilophilic Interaction toward Heme	Lilophilic Interaction with Phe−Cluster			
**Dihydrotestosterone**							
1	8.94	−	−	−	−28.671	10-Methyl	5-Hydroxylation
2	8.85	−	−	−	−27.4635	10-Methyl	
3	4.21	−	+	−	−26.3921	3	
4	2.6	−	+	−	−26.0321	13-Methyl	
5	8.29	−	+	−	−25.5539	3	
6	8.65	−	−	−	−24.963	7	
7	9.21	−	−	−	−24.1265	7	
8	9.44	−	−	−	−23.568	10-Methyl	
9	6.78	Arg372	+	−	−23.1156	5	
10	9.78	−	−	−	−22.2897	10-Methyl	
**Epitesterone**							
1	7.3	Leu210	+	+	−33.0614	6	6-Hydroxylation
2	3.46	−	−	−	−32.8545	3	
3	4.39	−	−	−	−32.4326	3	
4	4.39	heme	+	−	−31.9293	17	
5	7.5	−	−	−	−31.5436	6	
6	4.89	−	−	−	−30.6887	3	
7	4.92	heme	+	−	−29.7235	17	
8	8.52	Ile369 Leu483	−	−	−28.7048	10-Methyl	
9	5.04	−	−	−	−28.3235	3	
10	5.88	−	−	−	−27.6542	3	

Table S1. *Cont.*

Rank	Distance	Key Interaction Features			Chemscore	Predicted Metabolic Sites	Actual Metabolic Sites
		Hydrogen Bond Interaction	Lilophilic Interaction toward Heme	Lilophilic Interaction with Phe−Cluster			
**Eplerenone**							
1	1.85	Thr309	−	−	−35.7939	2	6,21-Hydroxylation
2	1.92	Thr309	−	−	−35.3216	2	
3	2.32	−	−	−	−34.6532	2	
4	2.05	Thr309	−	−	−34.1503	2	
5	3.75	−	−	−	−33.4281	2	
6	3.87	Ser119	+	−	−32.7908	3	
7	3.85	−	−	+	−32.597	21	
8	4.03	−	−	−	−32.4978	21	
9	4.36	−	−	+	−31.9885	21	
10	4.72	$H_2$0637 Thr637	−	−	−31.5642	6	
**Finasteride**							
1	4.72	Arg372	−	−	−34.3664	T-Butyl	T-Butyl-Oxidation
2	2.55	−	−	−	−33.5396	19	
3	2.01	−	+	−	−32.7063	3	
4	4.7	−	−	−	−32.6547	T-Butyl	
5	8.36	Arg105	−	−	−32.6214	13-Methyl	
6	4.01	−	−	−	−32.6018	4	
7	4.72	Arg105	−	−	−32.4325	T-Butyl	
8	4.93	−	−	−	−32.123	T-Butyl	
9	2.87	−	+	−	−31.8776	3	
10	4.47	−	−	−	−31.6314	19	

Table S1. *Cont.*

Rank	Distance	Key Interaction Features			Chemscore	Predicted Metabolic Sites	Actual Metabolic Sites
		Hydrogen Bond Interaction	Lilophilic Interaction toward Heme	Lilophilic Interaction with Phe−Cluster			
**Medroxyprogesterone**							
1	8.32	−	−	−	−30.3548	10-Methyl	1,2,6-Hydroxylation
2	3.25	H_2O623 Arg105	−	+	−29.7153	6	
3	9.23	−	−	−	−28.6432	13-Methyl	
4	9.76	−	−	−	−27.7032	10-Methyl	
5	9.56	−	−	−	−26.3215	10-Methyl	
6	4.04	−	+	−	−25.892	2	
7	3.97	−	+	−	−25.1325	2	
8	5.65	−	−	−	−24.3216	19	
9	4.32	−	−	−	−23.6879	23	
10	4.79	−	−	−	−22.3216	23	
**Metandienone**							
1	4.43	Arg372	+	−	−34.0604	3	6-Hydroxylation
2	9.32	−	−	−	−33.7855	7	
3	3.09	H_20637	−	+	−32.4879	13-Methyl	
4	3.38	H_20637	−	+	−32.0393	13-Methyl	
5	9.79	−	−	−	−31.6541	10-Methyl	
6	9.68	−	−	−	−30.9864	10-Methyl	
7	3.33	−	+	−	−30.2339	17-Methyl	
8	3.86	−	−	+	−30.1732	6	
9	10.23	−	−	−	−29.654	7	
10	5.19	−	+	−	−28.8697	17-Methyl	

Table S1. *Cont.*

Rank	Distance	Key Interaction Features			Chemscore	Predicted Metabolic Sites	Actual Metabolic Sites
		Hydrogen Bond Interaction	Lilophilic Interaction toward Heme	Lilophilic Interaction with Phe−Cluster			
**Methyltesterone**							
1	9.24	−	−	−	−35.3666	3	6-Hydroxylation
2	9.38	−	−	−	−33.3371	10-Methyl	
3	3.85	−	+	+	−32.7932	6	
4	9.79	−	−	−	−32.1546	13-Methyl	
5	4.18	−	+	+	−30.4565	6	
6	10.23	−	−	−	−29.7834	13-Methyl	
7	6.99	Arg372	+	−	−29.3065	2	
8	10.85	−	−	−	−28.8945	13-Methyl	
9	10.97	−	−	−	−28.2346	10-Methyl	
10	11.05	−	−	−	−27.5648	10-Methyl	
**Pregnenolone**							
1	1.98	Leu210	−	−	−35.0142	20	16-Hydroxylation
2	4.19	−	−	−	−34.7378	20	
3	3.03	$H_2$0637 Glu374	+	−	−34.4651	16	
4	9.85	−	−	−	−32.2739	13-Methyl	
5	4.69	Leu210	−	−	−30.8978	20	
6	8.79	−	−	−	−30.2537	13-Methyl	
7	8.35	−	−	−	−29.7865	10-Methyl	
8	9.36	−	−	−	−29.3425	10-Methyl	
9	10.03	−	−	−	−28.6545	7	
10	9.98	−	−	−	−27.8556	13-Methyl	

Table S1. *Cont.*

Rank	Distance	Key Interaction Features			Chemscore	Predicted Metabolic Sites	Actual Metabolic Sites
		Hydrogen Bond Interaction	Lilophilic Interaction toward Heme	Lilophilic Interaction with Phe−Cluster			
				**Progesterone**			
1	2.68	$H_2$0637	+	−	−37.0959	6	6,16-Hydroxylation
2	3.03	−	−	−	−36.9991	2	
3	3.59	−	−	−	−34.8775	3	
4	3.25	−	−	−	−33.7225	2	
5	3.29	−	−	−	−32.3576	3	
6	3.45	−	−	−	−30.9865	2	
7	3.65	−	−	−	−29.7685	2	
8	4.05	−	−	−	−28.6534	2	
9	3.86	−	−	−	−27.3456	2	
10	4.13	−	−	−	−26.4561	2	
				**Resibufagenin**			
1	3.71	Glu374	−	+	−35.2461	11	5-Hydroxylation
2	4.17	Arg105 Arg106	+	+	−34.0458	5	
3	4.35	Arg106	+	+	−33.7865	5	
4	5.04	Ser19	+	−	−33.485	3	
5	4.13	−	−	−	−33.3622	3	
6	5.21	Ser19	+	−	−33.2542	3	
7	5.95	Ser19 Arg105	+	−	−33.1563	3	
8	7.09	−	+	−	−33.0235	3	
9	2.62	−	−	−	−32.9837	19	
10	9.63	−	−	−	−32.4635	13-Methyl	

Table S1. *Cont.*

Rank	Distance	Key Interaction Features			Chemscore	Predicted Metabolic Sites	Actual Metabolic Sites
		Hydrogen Bond Interaction	Lilophilic Interaction toward Heme	Lilophilic Interaction with Phe−Cluster			
			**Stanazole**				
1	3.4	−	−	−	−28.0198	3	6-Hydroxylation
2	3.59	−	−	−	−27.6352	3	
3	7.62	−	−	−	−26.14738	10-Methyl	
4	7.95	−	−	−	−25.2125	10-Methyl	
5	4.1	−	+	+	−24.2746	6	
6	4.58	−	+	+	−23.565	6	
7	4.62	Ile301	+	+	−22.8542	6	
8	3.9	Arg373	+	−	−22.40254	2	
9	3.72	Arg373	+	−	−22.0412	2	
10	8.64	−	−	−	−21.2345	10-Methyl	
			**Taurochenodeoxycholic Acid**				
1	4.53	−	+	−	−36.4201	24	6-Hydroxylation
2	8.79	−	−	−	−35.3709	13-Methyl	
3	4.54	−	+	−	−34.9812	24	
4	4.85	−	+	−	−34.2304	24	
5	4.97	−	+	−	−33.9324	24	
6	4.25	Gl+481 heme	+	−	−32.9213	3	
7	3.73	Arg106 H_2O623 heme	+	+	−32.9118	6	
8	5.03	−	+	−	−32.3412	24	
9	5.08	−	+	−	−32.0265	24	
10	9.78	−	−	−	−31.7845	13-Methyl	

Table S1. *Cont.*

Rank	Distance	Key Interaction Features			Chemscore	Predicted Metabolic Sites	Actual Metabolic Sites
		Hydrogen Bond Interaction	Lilophilic Interaction toward Heme	Lilophilic Interaction with Phe−Cluster			
**Testosterone**							
1	2.74	heme	+	+	−31.4668	17	6-Hydroxylation
2	3.35	H_2O623	−	−	−31.419	6	
3		H_2O623,ser119	−	−	−31.1069	12	
4	4.41	H_2O623	−	−	−30.8546	6	
5	4.48	H_2O623,ser119	−	−	−30.4225	6	
6	3.25	−	+	+	−30.1354	17	
7	4.87	−	+	+	−29.9865	17	
8	5.6	H_2O623,ser119	−	−	−29.6526	12	
9	7.75	Leu 210	+	−	−28.7545	13-Methyl	
10	7.85	−	+	−	−28.2384	13-Methyl	
**4-ChlorodehydroMethyltesterone**							
1	1.95	−	+	−	−33.7054	3	6-Hydroxylation
2	2.02	−	+	−	−32.053	3	
3	2.51	−	+	−	−30.987	3	
4	2.47	Thr309	+	+	−29.3985	4	
5	8.36	−	−	−	−28.7045	7	
6	3.41	−	+	−	−27.697	2	
7	3.88	−	+	−	−25.8245	6	
8	4.22	Leu483	−	+	−25.7083	1	
9	3.07	−	+	−	−24.6328	4	
10	4.09	−	+	−	−24.0354	3	

Table S1. *Cont.*

Rank	Distance	Key Interaction Features: Hydrogen Bond Interaction	Key Interaction Features: Lilophilic Interaction toward Heme	Key Interaction Features: Lilophilic Interaction with Phe−Cluster	Chemscore	Predicted Metabolic Sites	Actual Metabolic Sites
**5β-Cholestone-3α-7α-12α-triol**							
1	2.3	H_2O637 H_2O619 Arg372	+	−	−31.2291	23	23,24,25-Hydroxylation
2	2.35	H_2O619 Arg372 heme	−	−	−31.2249	26	
3	2.35	H_2O619 Arg372	−	−	−28.7235	26	
4	3.77	H2O637 Arg372	+	−	−26.3245	23	
5	3.12	H_2O619 Arg372	−	−	−24.7414	24	
6	4.6	H_2O619 Arg372	−	+	−22.31	6	
7	4.98	H_2O619	−	+	−20.4653	6	
8	3.77	H_2O619 Arg372	−	−	−19.8546	25	
9	3.91	H_2O619 Arg372	+	−	−18.4625	23	
10	3.85	H_2O619 Arg372	+	−	−18.2354	23	
**Lilopristone**							
1	4.23	−	+	−	−44.2677	15	11-*N*-Demethylation
2	4.65	−	+	−	−44.1235	14	
3	4.82	−	+	−	−44.0582	16	
4	3.78	−	−	−	−43.911	17	
5	3.97	−	+	−	−43.6212	18	
6	4.25	−	+	−	−43.5963	11-N	
7	4.36	−	+	−	−43.2345	14	
8	4.48	−	+	−	−43.0872	14	
9	4.97	−	+	−	−42.8532	15	
10	5.32	−	+	−	−42.4637	15	

Table S1. *Cont.*

Rank	Distance	Key Interaction Features			Chemscore	Predicted Metabolic Sites	Actual Metabolic Sites
		Hydrogen Bond Interaction	Lilophilic Interaction toward Heme	Lilophilic Interaction with Phe−Cluster			
**Mestranol**							
1	2.09	−	+	−	−33.733	3-Methyl	3-Demethylation
2	2.35	−	+	−	−33.6543	3-Methyl	
3	2.26	−	−	−	−33.5205	18	
4	2.67	−	−	−	−33.154	18	
5	2.96	−	+	−	−32.745	3-Methyl	
6	8.67	−	−	−	−32.1497	13-Methyl	
7	2.44	−	H_2O637	−	−31.6227	15	
8	3.22	−	+	−	−31.054	3-Methyl	
9	3.68	−	+	−	−30.685	3-Methyl	
10	4.55	−	−	−	−30.1225	13-Methyl	
**Mifepristone**							
1	3.57	−	+	−	−38.6142	16	11-*N*-Demethylation
2	3.76	−	+	−	−38.0564	16	
3	3.17	−	+	−	−37.6844	11-N	
4	4.04	−	+	−	−36.4583	16	
5	2.3	−	+	−	−35.3137	2	
6	8.98	−	−	−	−33.9396	13-Methyl	
7	9.35	−	−	−	−33.7345	13-Methyl	
8	3.68	Leu210	+	−	−33.5876	3	
9	4.57	−	+	−	−32.9875	16	
10	4.97	−	+	−	−32.2564	16	

Table S2. Original parameter of docking data for the compounds from test set.

Rank	Distance	Key Interaction Features			Chemscore	Predicted Metabolic Sites	Actual Metabolic Sites
		Hydrogen Bond Interaction	Lilophilic Interaction toward Heme	Lilophilic Interaction with Phe−Cluster			
**Bufalin**							
1	2.02	Val240	−	−	−38.9729	24	3-Keto 5-Hydroxylation
2	4.05	$H_2$0619 Arg106 Ala305 Ile301	+	+	−35.4563	5	
3	4.54	$H_2$0619 Arg106 Ile301	+	+	−34.9685	5	
4	9.63	−	−	−	−34.2356	13-Methyl	
5	2.13	Val240	−	−	−33.6542	24	
6	2.24	−	−	−	−33.023	24	
7	2.88	−	−	−	−32.6452	24	
8	4.59	Arg372	−	+	−31.7943	14	
9	2.76	−	−	−	−30.6398	24	
10	3.59	Val240	−	−	−30.2567	24	
**Bufotalin**							
1	4.49	$H_2$0637 Arg106	+	+	−29.1787	5	1,5-Hydroxylation
2	5.46	heme	−	−	−28.6532	3	
3	3.38	Thr309 H_2O619	+	−	−28.2145	17	
4	3.32	Thr309	+	−	−27.5704	17	
5	3.45	Thr309 H_2O619	+	−	−26.9864	17	
6	8.95	−	−	−	−26.3265	13-Methyl	
7	3.69	Thr309 H_2O619	+	−	−25.6397	17	
8	8.78	−	−	−	−25.0231	13-Methyl	
9	9.65	−	−	−	−24.6897	7	
10	9.78	−	−	−	−23.864	13-Methyl	

Table S2. *Cont.*

Rank	Distance	Key Interaction Features			Chemscore	Predicted Metabolic Sites	Actual Metabolic Sites
		Hydrogen Bond Interaction	Lilophilic Interaction toward Heme	Lilophilic Interaction with Phe−Cluster			
**Cortisone**							
1	3.22	heme	−	−	−33.5642	21	6-Hydroxylation
2	3.44	heme	−	−	−33.5324	21	
3	9.89	−	−	−	−33.5162	10-Methyl	
4	3.35	−	−	−	−32.6373	21	
5	3.46	heme	−	−	−31.7865	21	
6	4.65	−	−	−	−31.1996	16	
7	5.63	−	−	−	−30.4117	20-O	
8	4.72	−	−	−	−29.9865	16	
9	4.12	−	−	+	−29.4542	6	
10	4.96	heme	−	−	−28.63	21	
**Epiandrosterone**							
1	9.83	−	−	−	−30.6879	10-Methyl	16-Hydroxylation
2	9.92	−	−	−	−29.9875	10-Methyl	
3	10.23	−	−	−	−29.3524	10-Methyl	
4	3.79	Ser119	+	−	−28.633	16	
5	4	Ser119	+	−	−28.265	16	
6	7.33	Ser119	+	+	−27.9834	6	
7	10.56	−	−	−	−27.6375	7	
8	10.58	−	−	−	−27.3425	7	
9	4.78	Arg372	−	+	−27.0084	13-Methyl	
10	10.65	−	−	−	−26.6425	10-Methyl	

Table S2. *Cont.*

Rank	Distance	Key Interaction Features			Chemscore	Predicted Metabolic Sites	Actual Metabolic Sites
		Hydrogen Bond Interaction	Lilophilic Interaction toward Heme	Lilophilic Interaction with Phe−Cluster			
**Fluticasone**							
1	2.31	−	−	+	−35.0291	17	17-Carboxylic acid formation
2	2.57	−	−	+	−34.6532	17	
3	3.22	−	−	+	−34.0261	17	
4	4.41	−	−	−	−33.8653	17	
5	4.57	−	−	+	−32.453	17	
6	4.35	−	−	−	−31.6789	17	
7	1.95	−	+	−	−30.2586	3	
8	4.21	−	−	−	−29.3524	3	
9	3.36	−	−	+	−28.7645	17	
10	4.22	−	+	−	−28.0362	3	
**Lithcholic Acid**							
1	5.43	Leu210	+	−	−32.6542	24	6-Hydroxylation
2	5.36	Leu210	+	−	−31.4532	24	
3	5.65	Ile301	−	−	−30.6523	24	
4	6.35	Ile301 Glu308	+	−	−30.0214	23	
5	6.62	Ile301 Glu308	−	−	−29.6532	23	
6	8.96	−	−	−	−28.3214	13-Methyl	
7	9.02	−	−	−	−27.9856	13-Methyl	
8	9.1	−	−	−	−27.2415	13-Methyl	
9	6.35	H_2O637 Gln484 Ser312	+	+	−26.654	19-Methyl	
10	7.23	Glu374 Arg105	+	+	−26.0355	6	

Table S2. *Cont.*

Rank	Distance	Key Interaction Features			Chemscore	Predicted Metabolic Sites	Actual Metabolic Sites
		Hydrogen Bond Interaction	Lilophilic Interaction toward Heme	Lilophilic Interaction with Phe−Cluster			
**Methylpredisolone**							
1	3.82	−	+	+	−30.1996	10-Methyl	6-Hydroxylation
2	3.46	−	+	+	−29.3672	10-Methyl	
3	5.42	−	−	−	−28.4329	1	
4	5.63	−	−	−	−27.8635	1	
5	4.63	Ile301 Glu308	+	+	−27.1689	6	
6	5.63	−	−	−	−26.7256	4	
7	4.87	Ile301 Glu308	−	+	−26.3985	6	
8	3.98	−	+	+	−25.4692	10-Methyl	
9	4	−	−	−	−23.8534	10-Methyl	
10	3.98	−	−	+	−23.233	10-Methyl	
**Prednisolone**							
1	1.98	Leu210	−	−	−35.0142	20-O	16-Hydroxylation
2	2.19	−	−	−	−34.8564	20-O	
3	3.03	$H_2$0637 Glu374	+	−	−34.4651	16	
4	8.69	−	−	−	−32.2739	13-Methyl	
5	8.74	−	−	−	−31.5642	13-Methyl	
6	3.03	Leu210	−	−	−30.7854	20-O	
7	8.96	−	−	−	−30.125	10-Methyl	
8	9.05	−	−	−	−29.6874	10-Methyl	
9	9.04	−	−	−	−28.6325	13-Methyl	
10	9.23	−	−	−	−28.3624	10-Methyl	

Table S2. *Cont.*

Rank	Distance	Key Interaction Features			Chemscore	Predicted Metabolic Sites	Actual Metabolic Sites
		Hydrogen Bond Interaction	Lilophilic Interaction toward Heme	Lilophilic Interaction with Phe−Cluster			
**Onapristone**							
1	5.38	Leu210	−	+	−42.8212	11-N	11-*N*-Demethylation
2	5.6	Ala370	−	−	−40.2616	15	
3	9.23	−	−	−	−40.137	10-Methyl	
4	5.65	−	−	+	−39.8452	11-N	
5	5.65	−	−	+	−39.2332	11-N	
6	5.9	Glu308	−	−	−39.6532	11-N	
7	4.19	Arg105 Glu374	−	+	−38.4142	6	
8	4.9	Arg105 Glu374	−	+	−38.0235	6	
9	9.78	−	−	−	−37.6524	10-Methyl	
10	4.83	Arg105	−	+	−36.632	6	
**Tirilazad**							
1	5.48	−	+	−	−53.0624	6	6-Hydroxylation
2	5.42	Ile301	+	−	−52.321	6	
3	4.14	−	−	−	−50.0319	16-Methyl	
4	2.97	−	+	−	−49.9946	4	
5	1.92	−	−	−	−49.917	3	
6	2.29	−	−	−	−49.6564	2	
7	3.58	−	−	−	−49.3625	3	
8	4.49	Arg372	+	−	−49.1728	12	
9	3.9	−	−	−	−48.6324	3	
10	5.49	−	+	−	−48.0234	4	

Table S2. *Cont.*

Rank	Distance	Key Interaction Features			Chemscore	Predicted Metabolic Sites	Actual Metabolic Sites
		Hydrogen Bond Interaction	Lilophilic Interaction toward Heme	Lilophilic Interaction with Phe−Cluster			
**Other Structure Alprazolam**							
1	2.84	Arg105	−	+	−33.2899	6	1,4-Hydroxylation
2	3.17	Arg105	−	+	−31.2568	6	
3	3.27	Ser119	+	−	−29.1964	1	
4	3.25	−	+	−	−28.6335	1	
5	3.38	−	+	−	−27.8324	1	
6	4.26	−	−	−	−27.1235	1	
7	3.42	Ser119	+	−	−26.3563	1	
8	4.86	Ser119	+	−	−25.9836	1	
9	4.75	−	−	−	−25.6321	1	
10	3.97	−	+	−	−24.5642	1	
**Clozapine**							
1	2.34	−	−	+	−26.5332	4	*N*-Oxidation
2	8.65	−	−	−	−26.0737	N	
3	2.28	−	−	+	−25.9836	4	
4	2.35	−	−	+	−25.9326	4	
5	3.25	−	−	+	−25.7325	4	
6	9.68	−	−	+	−25.2381	4	
7	9.75	−	−	−	−25.0317	N	
8	8.96	−	−	−	−24.9368	N	
9	9.48	−	−	−	−24.7328	N	
10	2.37	−	+	−	−24.6407	N	

Table S2. *Cont.*

Rank	Distance	Key Interaction Features			Chemscore	Predicted Metabolic Sites	Actual Metabolic Sites
		Hydrogen Bond Interaction	Lilophilic Interaction toward Heme	Lilophilic Interaction with Phe−Cluster			
**Flunitrazepam**							
1	5.73	Arg105	−	−	−26.1514	1	3-Hydroxylation *N*-Demethylation
2	5.73	Arg105	−	−	−25.3212	1	
3	3.54	Arg105	+	−	−24.8818	3	
4	3.46	Arg105	+	−	−24.2356	3	
5	5.72	Arg105	−	−	−23.6547	1	
6	5.95	−	+	−	−23.0615	1	
7	5.78	Arg105	−	−	−22.3214	1	
8	8.65	−	−	−	−21.9874	3	
9	5.83	−	+	−	−21.5632	3	
10	8.79	−	−	−	−20.3654	3	
**Ketamine**							
1	7.96	−	−	−	−27.4334	2	*N*-Demethylation
2	2.48	−	+	−	−27.0365	N	
3	2.33	−	+	−	−26.6897	2	
4	3.21	−	+	−	−26.3214	2	
5	8.06	−	−	−	−25.4368	2	
6	8.63	−	−	−	−24.7635	2	
7	8.45	−	−	−	−24.0321	2	
8	8.98	−	−	−	−23.306	N	
9	9.02	−	−	−	−23.2605	N	
10	5.69	−	−	+	−22.5102	2	

Table S2. *Cont.*

Rank	Distance	Key Interaction Features			Chemscore	Predicted Metabolic Sites	Actual Metabolic Sites
		Hydrogen Bond Interaction	Lilophilic Interaction toward Heme	Lilophilic Interaction with Phe−Cluster			
**LAAM**							
1	5.35	−	+	−	−32.8456	6	*N*-Demethylation
2	5.42	−	+	−	−31.4532	6	
3	5.38	−	−	−	−30.265	6	
4	2.82	−	−	+	−29.0164	3	
5	3.78	Ser19	−	+	−28.5253	3	
6	4.23	−	−	−	−28.1325	4	
7	3.93	−	−	−	−27.8652	4	
8	5.07	−	+	−	−27.6637	4	
9	5.36	−	−	−	−26.9836	4	
10	5.67	−	−	−	−26.3256	6	
**Midazolam**							
1	3.27	−	+	−	−26.6934	3	1,4-Hydroxylation
2	2.75	−	−	+	−26.3068	6	
3	2.88	−	−	+	−25.5423	6	
4	5.78	−	−	+	−24.8249	8−Chloro	
5	3.46	−	−	+	−24.503	3	
6	2.72	−	+	−	−24.0718	1	
7	2.97	−	+	−	−23.6423	1	
8	3.73	−	−	+	−23.1254	3	
9	3.56	−	+	−	−22.645	1	
10	4.18	−	+	−	−21.9863	1	

Table S2. *Cont.*

Rank	Distance	Key Interaction Features			Chemscore	Predicted Metabolic Sites	Actual Metabolic Sites
		Hydrogen Bond Interaction	Lilophilic Interaction toward Heme	Lilophilic Interaction with Phe−Cluster			
				**Quinidine**			
1	5.55	Ile301	−	+	−34.6414	3	3-Hydroxylation
2	5.54	Ile301	−	+	−33.2563	3	
3	5.65	Ile301	−	+	−32.6534	3	
4	5.72	Ile301	−	+	−31.2357	3	
5	3.86	−	+	−	−30.6583	N	
6	3.93	−	+	−	−30.1031	C	
7	4.59	−	+	−	−29.6875	N	
8	4.02	−	+	−	−29.3214	N	
9	4.23	−	+	−	−28.6573	N	
10	4.22	−	+	−	−27.3321	N	
				**Schizandrin A**			
1	5.36	Ser119	+	−	−34.3394	1	7-Hydroxylation
2	5.65	Ser119	+	−	−33.6345	1	
3	5.58	Ser119	−	−	−32.9785	1	
4	5.05	H_2O623	−	+	−32.1586	7	
5	5.55	H_2O623	−	+	−31.6325	7	
6	7.93	−	−	−	−30.7835	2	
7	8.32	−	−	−	−30.0244	2	
8	6.36	−	+	−	−29.6345	1	
9	8.49	−	−	−	−28.6378	2	
10	6.47	−	+	−	−28.4237	1	

Table S2. *Cont.*

Rank	Distance	Key Interaction Features			Chemscore	Predicted Metabolic Sites	Actual Metabolic Sites
		Hydrogen Bond Interaction	Lilophilic Interaction toward Heme	Lilophilic Interaction with Phe−Cluster			
**Schizandrin B**							
1	2.12	−	+	−	−26.5975	1	7-Hydroxylation
2	3.18	$H_2$0623 Ile 369	−	−	−25.1363	12	
3	3.09	$H_2$0623	−	−	−24.6358	12	
4	8.05	−	−	−	−24.1121	2	
5	7.98	−	−	−	−23.9874	2	
6	3.89	$H_2$0637	−	+	−23.4794	7	
7	4.7	−	+	−	−22.7865	1	
8	3.85	−	+	−	−22.5632	1	
9	4.23	−		−	−22.2341	1	
10	4.08	−	+	−	−21.745	1	
**Toremifene**							
1	4.71	−	−	+	−42.1659	4-Chloro	2-Hydroxylation *N*-Demethylation
2	8.4	−	−	−	−41.5638	2-Phenoxy	
3	4.92	−	−	+	−40.7652	4-Chloro	
4	4.89	−	−	+	−40.6321	4-Chloro	
5	8.23	−	−	−	−40.0325	2-Phenoxy	
6	8.56	−	−	−	−39.6985	2-Phenoxy	
7	8.98	−	−	−	−39.2312	2-Phenoxy	
8	4.68	−	+	−	−38.4699	2-Phenoxy	
9	4.61	−	+	−	−38.35	N	
10	6.4	−	−	+	−37.9865	4-Chloro	

Table S2. *Cont.*

Rank	Distance	Key Interaction Features			Chemscore	Predicted Metabolic Sites	Actual Metabolic Sites
		Hydrogen Bond Interaction	Lilophilic Interaction toward Heme	Lilophilic Interaction with Phe−Cluster			
				**Triazolam**			
1	10.32	−	−	−	−26.3366	4	1,4-Hydroxylation
2	4.49	−	+	−	−24.1722	3	
3	4.72	−	+	−	−24.1308	3	
4	3.01	−	+	−	−24.0811	1	
5	10.18	−	−	−	−23.6324	4	
6	10.65	−	−	−	−22.987	4	
7	4.68	−	+	−	−22.2654	3	
8	4.93	−	+	−	−21.5665	3	
9	3.2	−	+	−	−21.0235	1	
10	5.15	−	−	−	−20.6452	3	
				**Zotepine**			
1	3.45	−	−	−	−37.1058	2	S-Oxidation *N*-Demethylation
2	7.93	−	−	−	−36.5328	2	
3	3.49	−	−	−	−35.7356	2	
4	3.36	Arg372	−	+	−35.1572	S	
5	8.15	−	−	−	−34.2315	S	
6	8.09	−	−	−	−33.0238	2	
7	8.23	−	−	−	−31.5192	2	
8	3.78	−	−	+	−31.2638	S	
9	3.82	Arg372	−	+	−31.0872	S	
10	4.01	−	+	−	−30.9275	N	

Distance, the distances (*r*) between the heme iron of CYP3A4 and the atoms of the substrates; +, The lilophilic interaction (toward heme or with phe−cluster) was formed in the prediction of substrate-enzyme interaction; −, No hydrogen bond interaction or lilophilic interaction was formed in the prediction of substrate-enzyme interaction.
